# The photon PDF from high-mass Drell–Yan data at the LHC

**DOI:** 10.1140/epjc/s10052-017-4931-5

**Published:** 2017-06-15

**Authors:** F. Giuli, and the xFitter Developers’ team, V. Bertone, D. Britzger, S. Carrazza, A. Cooper-Sarkar, A. Glazov, K. Lohwasser, A. Luszczak, F. Olness, R. Plačakytė, V. Radescu, J. Rojo, R. Sadykov, P. Shvydkin, O. Zenaiev, M. Lisovyi

**Affiliations:** 1 University of Oxford, 1 Keble Road, Oxford, OX1 3NP UK; 2 Department of Physics and Astronomy, VU University, 1081 HV Amsterdam, The Netherlands; 3 Nikhef Theory Group Science Park 105, 1098 XG Amsterdam, The Netherlands; 4 DESY Hamburg, Notkestrasse 85, 22609 Hamburg, Germany; 5 CERN, 1211 Geneva 23, Switzerland; 6 DESY Zeuthen, Platanenallee 6, 15738 Zeuthen, Germany; 7 T. Kosciuszko Cracow University of Technology, 30-084 Cracow, Poland; 8 SMU Physics, Box 0175, Dallas, TX 75275-0175 USA; 9 Institut für Theoretische Physik, Universität Hamburg, Luruper Chaussee 149, 22761 Hamburg, Germany; 10 Joint Institute for Nuclear Research (JINR), Joliot-Curie 6, 141980 Dubna, Moscow Region Russia; 11 Physikalisches Institut, Ruprecht-Karls-Universität Heidelberg, Heidelberg, Germany

## Abstract

Achieving the highest precision for theoretical predictions at the LHC requires the calculation of hard-scattering cross sections that include perturbative QCD corrections up to (N)NNLO and electroweak (EW) corrections up to NLO. Parton distribution functions (PDFs) need to be provided with matching accuracy, which in the case of QED effects involves introducing the photon parton distribution of the proton, $x\gamma (x,Q^2)$. In this work a determination of the photon PDF from fits to recent ATLAS measurements of high-mass Drell–Yan dilepton production at $\sqrt{s}=8$ TeV is presented. This analysis is based on the xFitter framework, and has required improvements both in the APFEL program, to account for NLO QED effects, and in the aMCfast interface to account for the photon-initiated contributions in the EW calculations within MadGraph5_aMC@NLO. The results are compared with other recent QED fits and determinations of the photon PDF, consistent results are found.

## Introduction

Precision phenomenology at the LHC requires theoretical calculations which include not only QCD corrections, where NNLO is rapidly becoming the standard, but also electroweak (EW) corrections, which are particularly significant for observables directly sensitive to the TeV region, where EW Sudakov logarithms are enhanced. An important ingredient of these electroweak corrections is the photon parton distribution function (PDF) of the proton, $x\gamma (x,Q^2)$, which must be introduced to absorb the collinear divergences arising in initial-state QED emissions.

The first PDF fit to include both QED corrections and a photon PDF was MRST2004QED [1], where the photon PDF was taken from a model and tested on HERA data for direct photon production. Almost 10 years later, the NNPDF2.3QED analysis [2, 3] provided a first model-independent determination of the photon PDF based on Drell–Yan (DY) data from the LHC. The resulting photon PDF was, however, affected by large uncertainties due to the limited sensitivity of the data used as input to that fit. The determination of $x\gamma (x,Q^2)$ from NNPDF2.3QED was later combined with the state-of-the-art quark and gluon PDFs from NNPDF3.0, together with an improved QED evolution, to construct the NNPDF3.0QED set [4, 5]. The CT group has also released a QED fit using a similar strategy as the MRST2004QED one, named the CT14QED set [6].

A recent breakthrough concerning the determination of the photon content of the proton has been the realisation that $x\gamma (x,Q^2)$ can be calculated in terms of inclusive lepton–proton deep-inelastic scattering (DIS) structure functions. The photon PDF resulting from this strategy is called LUXqed [7] and its residual uncertainties are now at the few percent level, not too different from those of the quark and gluon PDFs. A related approach by the HKR [8] group, denoted by HKR16 in the following, also leads to a similar photon PDF as compared to the LUXqed calculation, although in this case no estimate for the associated uncertainties is provided.

The aim of this work is to perform a direct determination of the photon PDF from recent high-mass Drell–Yan measurements from ATLAS at $\sqrt{s}=8$ TeV [9], and to compare it with some of the existing determinations of $x\gamma (x,Q^2)$ mentioned above. Note that earlier measurements of high-mass DY from ATLAS and CMS were presented in Refs. [10–12]. The ATLAS 8 TeV DY data are provided in terms of both single-differential cross-section distributions in the dilepton invariant mass, $m_{ll}$, and of double-differential cross-section distributions in $m_{ll}$ and $|y_{ll}|$, the absolute value of rapidity of the lepton pair, and in $m_{ll}$ and $\Delta \eta _{ll}$, the difference in pseudo-rapidity between the two leptons. Using the Bayesian reweighting method [13, 14] applied to NNPDF2.3QED, it was shown in the same publication [9] that these measurements provided significant information on $x\gamma (x,Q^2)$.

The goal of this study is therefore to investigate further these constraints from the ATLAS high-mass DY measurements on the photon PDF, this time by means of a direct PDF fit performed within the open-source xFitter framework [15]. State-of-the-art theoretical calculations are employed, in particular the inclusion of NNLO QCD and NLO QED corrections to the PDF evolution and the computation of the DIS structure functions as implemented in the APFEL program [16]. The implementation of NLO QED effects in APFEL is presented here for the first time. The inclusion of NLO QED evolution effects is cross-checked using the independent QEDEVOL code [17] based on the QCDNUM evolution program [18].

The resulting determination of $x\gamma (x,Q^2)$ represents an important validation test of recent developments in theory and data concerning our understanding of the nature and implications of the photon PDF.

The outline of this paper is as follows. Section 2 reviews the ATLAS 8 TeV high-mass DY data together with the theoretical formalism of the DIS and Drell–Yan cross sections used in the analysis. Section 3 presents the settings of the PDF fit within the xFitter framework. The fit results are then discussed in Sect. 4, where they are compared to determinations by other groups. Finally, Sect. 5 summarises and discusses the results and future lines of investigation. “Appendix A” contains a detailed description of the implementation and validation of NLO QED corrections to the DGLAP PDF evolution equations and DIS structure functions, which are available now in APFEL.

## Data and theory

**Fig. 1:**
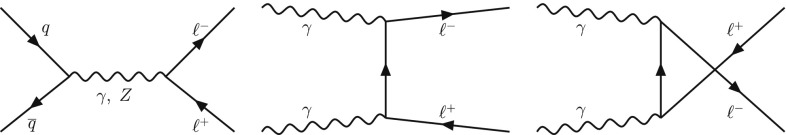
Diagrams that contribute to lepton-pair production at hadron colliders at the Born level

In this work, the photon content of the proton $x\gamma (x,Q^2)$ is extracted from a PDF analysis based on the combined inclusive DIS cross-section data from HERA [19] supplemented by the ATLAS measurements of high-mass Drell–Yan differential cross sections at $\sqrt{s}=8$ TeV [9]. The HERA inclusive data are the backbone of modern PDF fits, providing information on the quark and gluon content of the proton, while the high-mass Drell–Yan data provide a direct sensitivity to the photon PDF. As illustrated in Fig. 1, dilepton production at hadron colliders can arise from either quark–antiquark *s*-channel scattering, or from photon–photon *t*- and *u*-channel scattering mediated by a lepton.

The ATLAS high-mass Drell–Yan 8 TeV measurements are presented in terms of both the single-differential (1D) invariant-mass distribution, $$\mathrm{{d}}\sigma /\mathrm{{d}}m_{ll}$$, and the double-differential (2D) distributions in $$m_{ll}$$ and $$y_{ll}$$, namely $$\mathrm{{d}}^{2}\sigma /\mathrm{{d}}m_{ll}\mathrm{{d}}|y_{ll}|$$, and in $$m_{ll}$$ and $$\Delta \eta _{ll}$$, $$\mathrm{{d}}^{2}\sigma /\mathrm{{d}}m_{ll}\Delta \eta _{ll}$$. For the invariant-mass 1D distribution, there are 12 bins between $$m_{ll}=116$$ GeV and 1.5 TeV; and for both double-differential distributions, there are five different bins in invariant mass, from the lowest bin with 116 GeV $$< m_{ll} < $$150 GeV to the highest bin with 500 GeV $$< m_{ll}<$$ 1500 GeV.

The first three (last two) $m_{ll}$ bins of the 2D distributions are divided into 12 (6) bins with fixed width, extending up to 2.4 and 3.0 for the $|y_{ll}|$ and $|\Delta \eta _{ll}|$ distributions, respectively.

The photons which undergo hard scattering in the $\gamma \gamma \rightarrow ee$ process from Fig. 1 can be produced by either emission from the proton as a whole (the “elastic” component) or radiated by the constituent quarks (the “inelastic” component). From the theory point of view, the photon PDF extracted from the fit is by construction the sum of the elastic and inelastic contributions.

For the calculation of NLO high-mass Drell–Yan cross sections, the MadGraph5 _aMC@NLO [20] program is used, which includes the contribution from photon-initiated diagrams, interfaced to APPLgrid [21] through aMCfast [22]. A tailored version of APPLgrid is used, accounting for the contribution of the photon-initiated processes.^1^ The calculation is performed in the $n_f=5$ scheme neglecting mass effects of charm and bottom quarks in the matrix elements, as appropriate for a high-scale process. These NLO theoretical predictions match the analysis cuts of the data, with $m_{ll}\ge 116$ GeV, $\eta _l\le 2.5$, and $p_T^l \ge 40$ GeV (30) GeV for the leading (sub-leading) lepton being the most important ones. As discussed below, the NLO calculations are then supplemented by NNLO/NLO *K*-factors obtained from FEWZ [23]. The NLO EW corrections to the DY processes are also estimated using FEWZ. The photon-initiated process is taken at LO since this corresponds to the APPLgrid implementation and the NLO corrections are very small compared to the data accuracy.

The DIS structure functions and PDF evolution are computed with the APFEL program [16], which is currently accurate up to NNLO in QCD and NLO in QED, including the relevant mixed QCD + QED corrections. This means that, on top of the pure QCD contributions, the DGLAP evolution equations [24–26] are solved including the $\mathscr {O}\left( \alpha _s\alpha \right)$ and $\mathscr {O}\left( \alpha ^2\right)$ corrections to the splitting functions. Corrections of $\mathscr {O}\left( \alpha \right)$ are also included leading to a (weak) explicit dependence of the predictions on the photon PDF. Details of the implementation of these corrections and of their numerical impact are given in “Appendix A”. Heavy-quark (charm and bottom) mass effects to DIS structure functions are taken into account using the FONLL-B (C) general-mass scheme [27] for the NLO (NNLO) fits. The numerical values of the heavy-quark masses in the mass parameter scheme are taken to be $m_c=1.47~$GeV and $m_b=4.5~$GeV as determined in [19], consistent with the latest PDG averages [28]. The reference values of the QCD and QED coupling constants are chosen to be $\alpha _s(m_Z)=0.118$ and $\alpha (m_\tau =1.777 \text{ GeV })=1/133.4$, again consistent with the PDG recommended values.

In the calculation of the Drell–Yan cross section, the dynamical renormalisation $\mu _{R}$ and factorisation $\mu _{F}$ scales are used, which are set equal to the scale of invariant mass $m_{ll}$, both for the quark- and gluon-induced and for the photon-induced contributions. The choice of other values for these scales in the QED diagrams, such as a fixed scale $\mu _R=\mu _F=M_Z$, leads to variations of the photon-initiated cross sections of at most a few percent. The choice of the scale for the photon PDF is further discussed in [29, 30]. For the kinematics of the ATLAS DY data, the ratio between the photon-initiated contributions and quark- and gluon-induced dilepton production is largest for central rapidities and large invariant masses. For the most central (forward) rapidity bin, $0< |y_{ll}| < 0.2$ ($2.0< |y_{ll}| < 2.4$), the ratio between the QED and QCD contributions varies between 2.5% (2%) at low invariant masses and 12% (2.5%) for the highest $m_{ll}$ bin, based on MMHT14nnlo_68cl PDF set for the QCD contribution and NNPDF30qed_nnlo_as_0118 PDF set for the LO QED contributions. Therefore, data from the central region will exhibit the highest sensitivity to $x\gamma (x,Q^2)$.

The MadGraph5_aMC@NLO NLO QCD and LO QED calculations used in this work have been benchmarked against the corresponding predictions obtained with the FEWZ code [23], finding agreement within statistical uncertainties of the predictions for both the 1D and the 2D distributions.

**Fig. 2:**
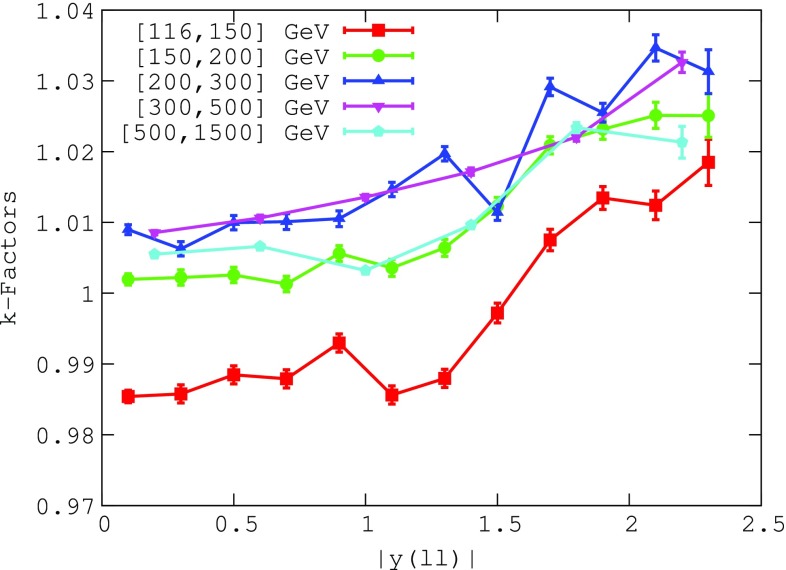
The NNLO/NLO *K*-factors, defined in Eq. (1), which account for higher-order QCD and EW effects to the high-mass Drell–Yan cross sections with the photon-induced contribution subtracted, as a function of the dilepton rapidity $|y_{ll}|$. Each set of points corresponds to a different bin in the dilepton invariant mass $m_{ll}$

In order to achieve NNLO QCD and NLO EW accuracy in our theoretical calculations, the NLO QCD and LO QED cross sections computed with MadGraph5_aMC@NLO have been supplemented by bin-by-bin *K*-factors defined by

1 $$\begin{aligned} K(m_{ll},|y_{ll}|) \equiv \frac{{\mathrm{NNLO}}\,\,{\mathrm{QCD}}+ {\mathrm{NLO}}\,\,{\mathrm{EW}}}{\mathrm{NLO}\,\, \mathrm{QCD + LO\,\, EW}}, \nonumber \\ \end{aligned}$$

using the MMHT2014 NNLO [31] PDF set both in the numerator and in the denominator. The *K*-factors have been computed using FEWZ with the same settings and analysis cuts as the corresponding NLO calculations of MadGraph5_aMC@NLO. This approximation is justified since the NNLO *K*-factors as defined in Eq. (1) depend very mildly on the input PDF set; see for example Ref. [32]. The photon-induced contribution, as provided in Ref. [9], has been explicitly subtracted from the FEWZ predictions. Figure 2 shows the *K*-factors of Eq. (1) corresponding to the double-differential $(m_{ll},|y_{ll}|)$ cross sections as a function of the dilepton rapidity $|y_{ll}|$, where each set of points corresponds to a different dilepton invariant mass $m_{ll}$ bin. The *K*-factors vary between 0.98 and 1.04, highlighting the fact that higher-order corrections to the Drell–Yan process are moderate, in particular at low values of $m_{ll}$ and in the central region. Even at forward rapidities, such *K*-factors modify the NLO QCD + LO EW results by at most 4%. Based on the definition in Eq. (1), in the rest of the paper, unless explicitly specified, we will refer to NNLO by actually meaning NNLO in QCD plus NLO EW corrections, and to NLO by meaning NLO in QCD plus LO EW corrections.

## Settings

This section presents the settings of the PDF fits, including the details of the parametrisation of the photon PDF $x\gamma (x,Q^2)$, which have been carried out using the open-source xFitter framework [15]. First of all, the scale $Q_0^2$ at which PDFs are parametrised is taken to be $Q_0^2 = 7.5~$GeV$^2$, which coincides with the value $Q_\mathrm{min}^2$ that defines the kinematic cut $Q^2 \ge Q_\mathrm{min}^2$ for the data points that are used as input to the fits. The charm PDF is then generated perturbatively from quarks and gluons by means of DGLAP evolution, exploiting recent developments in APFEL which allow the setting of heavy-quark thresholds $\mu _h$ differently from the heavy-quark masses $m_h$, such that $\mu _c=Q_0 > m_c$. Hence a high threshold can be used without having to parametrise the charm PDF [33].

The expression for the $\chi ^2$ function used for the fits is that of Ref. [34], which includes corrections for possible biases from statistical fluctuations and treats the systematic uncertainties multiplicatively. Alternative forms that do not include these corrections, such as those defined in [19, 35], have also been studied, but no significant differences in the results have been observed.

In this analysis, the parametrised PDFs are the valence distributions $xu_{v}(x,Q_0^2)$ and $xd_{v}(x,Q_0^2)$, the gluon distribution $xg(x,Q_0^2)$, and the *u*-type and *d*-type sea-quark distributions, $x\bar{U}(x,Q_0^2)$, $x\bar{D}(x,Q_0^2)$, where $x\bar{U}(x,Q_0^2) = x\bar{u}(x,Q_0^2)$ and $x\bar{D}(x,Q_0^2) = x\bar{d}(x,Q_0^2) + x\bar{s}(x,Q_0^2)$. In addition, the photon distribution $x\gamma (x,Q_0^2)$ is also parametrised at the starting scale. The following general functional form is adopted:

2 $$\begin{aligned} xf(x, Q_0^2) = Ax^{B}(1-x)^{C}(1+Dx+Ex^{2}) \, , \end{aligned}$$

where some of the normalisation parameters, in particular $A_{u_{v}}$, $A_{d_{v}}$ and $A_{g}$, are constrained by the valence and momentum sum rules (note that the photon PDF also enters the momentum sum rule). The parameters $B_{\bar{U}}$ and $B_{\bar{D}}$ are set equal to each other, so that the two quark sea distributions share a common small-*x* behaviour. Since the measurements used here are not sensitive to the strangeness content of the proton, strangeness is fixed such that $x\bar{s} (x, Q_0^2) = r_sx\bar{d}(x,Q_0^2)$, where $r_s=1.0$ is consistent with the ATLAS analysis of inclusive *W* and *Z* production [36, 37]. The further constraint $A_{\bar{U}} = 0.5 A_{\bar{D}}$ is imposed, such that $x\bar{u}(x,Q_0^2) \rightarrow x\bar{d}(x,Q_0^2)$ as $x \rightarrow 0$.

The explicit form of the PDF parametrisation Eq. (2) at the scale $Q_0^2$ is determined by the technique of saturation of the $\chi ^{2}$, namely the number of parameters is increased one by one until the $\chi ^{2}$ does not improve further, employing Wilks’ theorem [38]. Following this method, the optimal parametrisation for the quark and gluon PDFs found for this analysis is

3 $$\begin{aligned} xu_v(x)= & {} A_{u_v}x^{B_{u_v}}(1-x)^{C_{u_v}}(1+E_{u_v}x^{2})\, , \nonumber \\ xd_v(x)= & {} A_{d_v}x^{B_{d_v}}(1-x)^{C_{d_v}}\, , \nonumber \\ x\bar{U}(x)= & {} A_{\bar{U}}x^{B_{\bar{U}}}(1-x)^{C_{\bar{U}}}\, , \nonumber \\ x\bar{D}(x)= & {} A_{\bar{D}}x^{B_{\bar{D}}}(1-x)^{C_{\bar{D}}}\, ,\nonumber \\ xg(x)= & {} A_{g}x^{B_{g}}(1-x)^{C_{g}}(1+E_{g}x^{2})\, , \end{aligned}$$

while for the photon PDF it is used:

4 $$\begin{aligned} x\gamma (x) = A_{\gamma }x^{B_{\gamma }}(1-x)^{C_{\gamma }}(1+D_{\gamma }x+E_{\gamma }x^{2}). \end{aligned}$$

The parametrisation of the quark and gluon PDFs in Eq. (3) differs from the one used in the HERAPDF2.0 analysis in various ways. First of all, a higher value of the input evolution scale $Q_0^2$ is used, which is helpful to stabilise the fit of the photon PDF. Second, an additional negative term in the parametrisation of the gluon is not required here, because of the increased value of $Q_0^2$ which ensures the positiveness of the gluon distribution. Third, the results of the parametrisation scan are different because of the inclusion of the ATLAS high-mass Drell–Yan cross-section data.

PDF uncertainties are estimated using the Monte Carlo replica method [39–41], cross-checked with the Hessian method [42] using $\Delta \chi ^2=1$. The former is expected to be more robust than the latter, due to the potential non-Gaussian nature of the photon PDF uncertainties [3]. In Sect. 4 it is shown that these two methods to estimate the PDF uncertainties on the photon PDF lead to similar results.

In addition, a number of cross-checks have been performed to assess the impact of various model and parametrisation uncertainties. For the model uncertainties, variations of the charm mass between $m_c=1.41$ to 1.53 GeV, of the bottom mass between $m_c=4.25$ to 4.75 GeV, of the strong coupling constant $\alpha _s(m_Z)$ between 0.116 to 0.120 are considered, and additionally the strangeness fraction is decreased down to $r_s=0.75$. For the parametrisation uncertainties, the impact of increasing the input parametrisation scale up to $Q_0^2=10$ GeV$^2$ is considered as well as the impact of including additional parameters in Eq. (3). These extra parameters make little difference to the $\chi ^2$ of the fit, but they can change the shape of the PDFs in a non-negligible way. Such additional parameters are $D_{u_v}$, $D_{\bar{u}}$, $E_{\bar{d}}$, as well and the extra negative term in the gluon PDF used in HERAPDF2.0. The impact of these model and parametrisation uncertainties on the baseline results is quantified in Sect. 4.3.

## Results

In this section the determination of the PDFs from a fit to HERA inclusive structure functions and ATLAS high-mass Drell–Yan cross sections, with an emphasis on the photon PDF is presented. First the fit quality is assessed and the fit results are compared with the experimental data. Then the resulting photon PDF is shown and compared with other recent determinations. The impact of the high-mass DY data on the quark PDFs is also quantified. Thus, the robustness of the fits of $x\gamma (x,Q^2)$ with respect to varying the model, parametrisation, and procedural inputs is assessed. Finally, perturbative stability is addressed by comparing NLO and NNLO results.

### Fit quality and comparison between data and fit results

In the following, the results that will be shown correspond to those obtained from fitting the double-differential $\left( m_{ll},y_{ll}\right)$ cross-section distributions. It has been verified that comparable results are obtained if the $\left( m_{ll},\Delta \eta _{ll}\right)$ cross-section distributions are fitted instead.

For the baseline NNLO fit, the value $\chi ^2_\mathrm{min}/N_\mathrm{dof} = 1284/1083$ is obtained where $N_\mathrm{dof}$ is the number of degrees of freedom in the fit which is equal to total number of data points minus number of free parameters. The contribution from the HERA inclusive data is $\chi ^{2}/N_\mathrm{dat} = 1236/1056$ and from the ATLAS high-mass DY data is $\chi ^2/N_\mathrm{dat} = 48/48$, where $N_\mathrm{dat}$ the number of the data points for the corresponding data sample. These values for $\chi ^{2}/N_\mathrm{dat}$, together with the corresponding values for the various invariant mass $m_{ll}$ bins of the ATLAS data, are summarised in Table 1. The quality of the agreement with the HERA cross sections is of comparable quality to that found in the HERAPDF2.0 analysis. Note that in the calculation of the total $\chi ^2$ for the ATLAS data, the correlations between the different $m_{ll}$ bins have been taken into account.

**Table 1:** The $\chi ^{2}/N_\mathrm{dat}$ in the NNLO fits for the HERA inclusive structure functions and for the various invariant mass $m_{ll}$ bins of the ATLAS high-mass DY data. In the latter case, the contribution to the $\chi ^2$ arising from the correlated and log-penalty terms are indicated, as well as the overall $\chi ^2/N_\mathrm{dof}$ is provided, where $N_\mathrm{dof}$ is the number of degree of freedom in the fit

Dataset	$\chi ^2$ /$N_\mathrm{dat}$
HERA I + II	1236/1056
High-mass DY 116 GeV $\le m_{ll} \le$ 150 GeV	9/12
High-mass DY 150 GeV $\le m_{ll} \le$ 200 GeV	15/12
High-mass DY 200 GeV $\le m_{ll} \le$ 300 GeV	14/12
High-mass DY 300 GeV $\le m_{ll} \le$ 500 GeV	5/6
High-mass DY 500 GeV $\le m_{ll} \le$ 1500 GeV	4/6
Correlated (high-mass DY) $\chi ^2$	1.17
Log penalty (high-mass DY) $\chi ^2$	$-$0.12
Total (high-mass DY) $\chi ^2/N_\mathrm{dat}$	48/48
Combined HERA I + II and high-mass DY $\chi ^2/N_\mathrm{dof}$	1284/1083

**Fig. 3:**
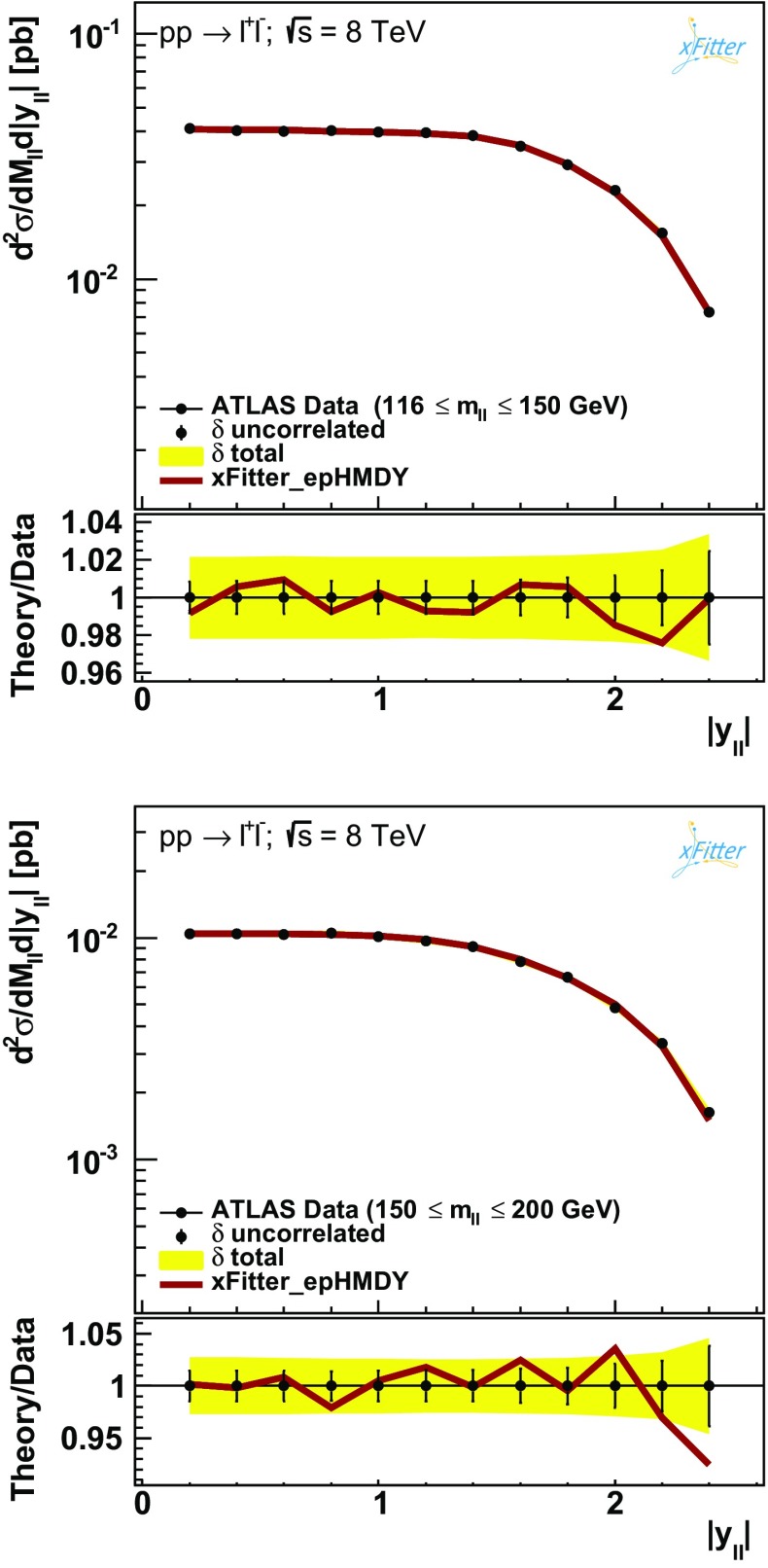
Comparison between the results of the fit and the ATLAS data for the $(m_{ll},|y_{ll}|)$ double-differential Drell–Yan cross sections as functions of $|y_{ll}|$, for the first two $m_{ll}$ bins. The comparisons are shown both in an absolute scale (*upper plots*) and as ratios to the central value of the experimental data in each $y_{ll}$ bin (*lower plots*). The *error bars* on the data points correspond to the bin-to-bin uncorrelated uncertainties, while the *yellow* bands indicate the size of the correlated uncertainties. The *solid lines* indicate the theory calculations obtained using the results of the fit xFitter_epHMDY

**Fig. 4:**
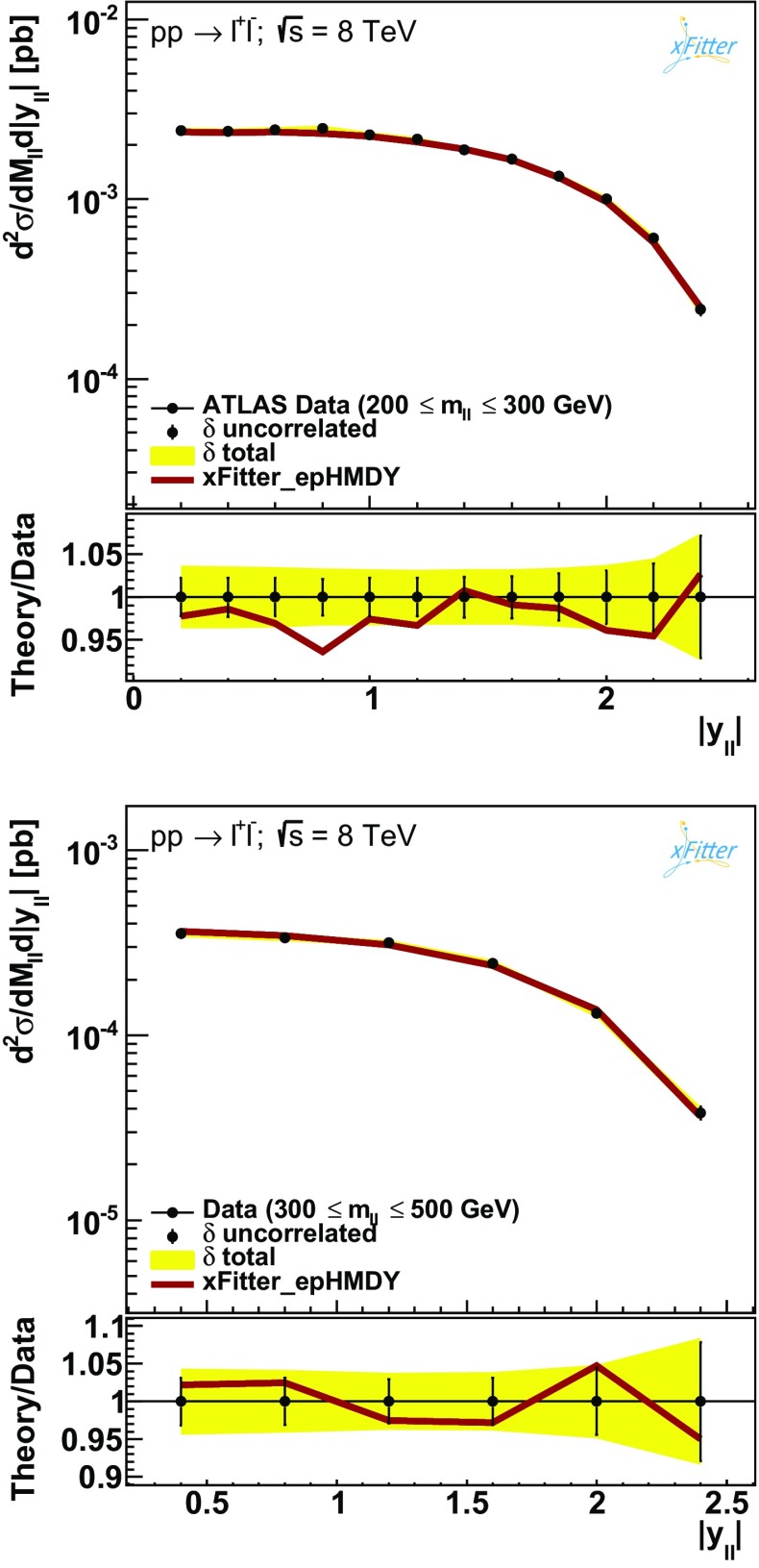
Same as Fig. 3 for the third and fourth $m_{ll}$ bins

**Fig. 5:**
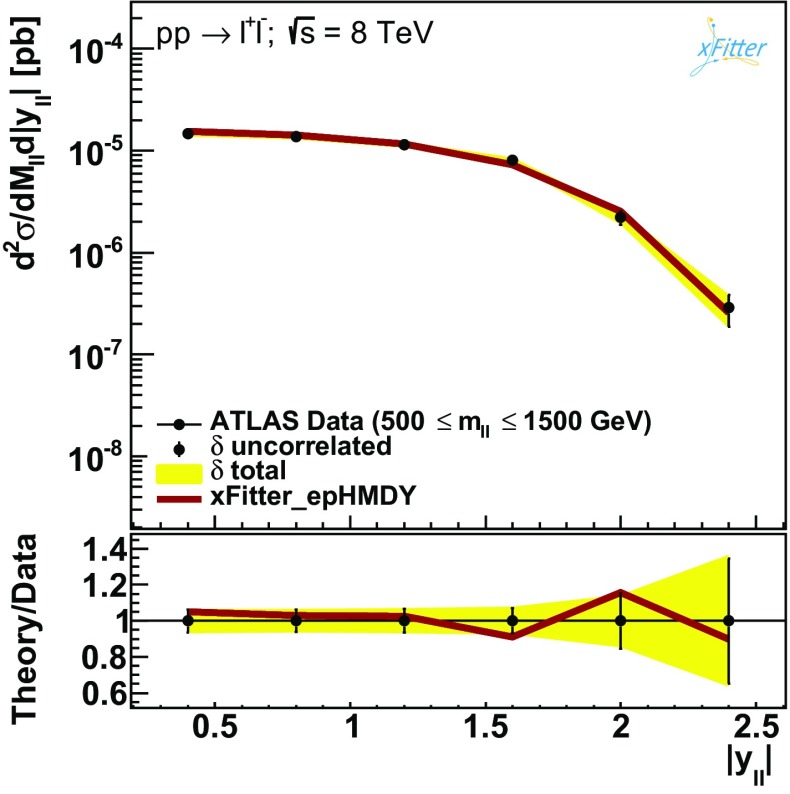
Same as Fig. 3 for the highest $m_{ll}$ bin

Figures 3, 4 and 5 then show the comparison between the results of the NNLO fit, denoted by xFitter_epHMDY, and the ATLAS data for the $(m_{ll},|y_{ll}|)$ double-differential Drell–Yan cross sections as functions of $|y_{ll}|$, for the five bins in $m_{ll}$ separately.

The comparisons are shown both on an absolute scale and as ratios to the central value of the experimental data.

The error bars on the data points correspond to the bin-to-bin uncorrelated uncertainties, while the bands indicate the size of the correlated systematic uncertainties.

The solid lines indicate the theory calculations obtained using the results of the fit.

Figures 3, 4 and 5 demonstrate good agreement between ATLAS data and the NNLO theory predictions obtained from the xFitter_epHMDY fit. This agreement is also quantitatively expressed by the values of the $\chi ^2$ reported in Table 1, where for the ATLAS data a $\chi ^2/N_\mathrm{dat}=1$ is found. This is particularly remarkable given the high precision of the data, with total experimental uncertainties at the few percent level in most of the kinematic range.

**Fig. 6:**
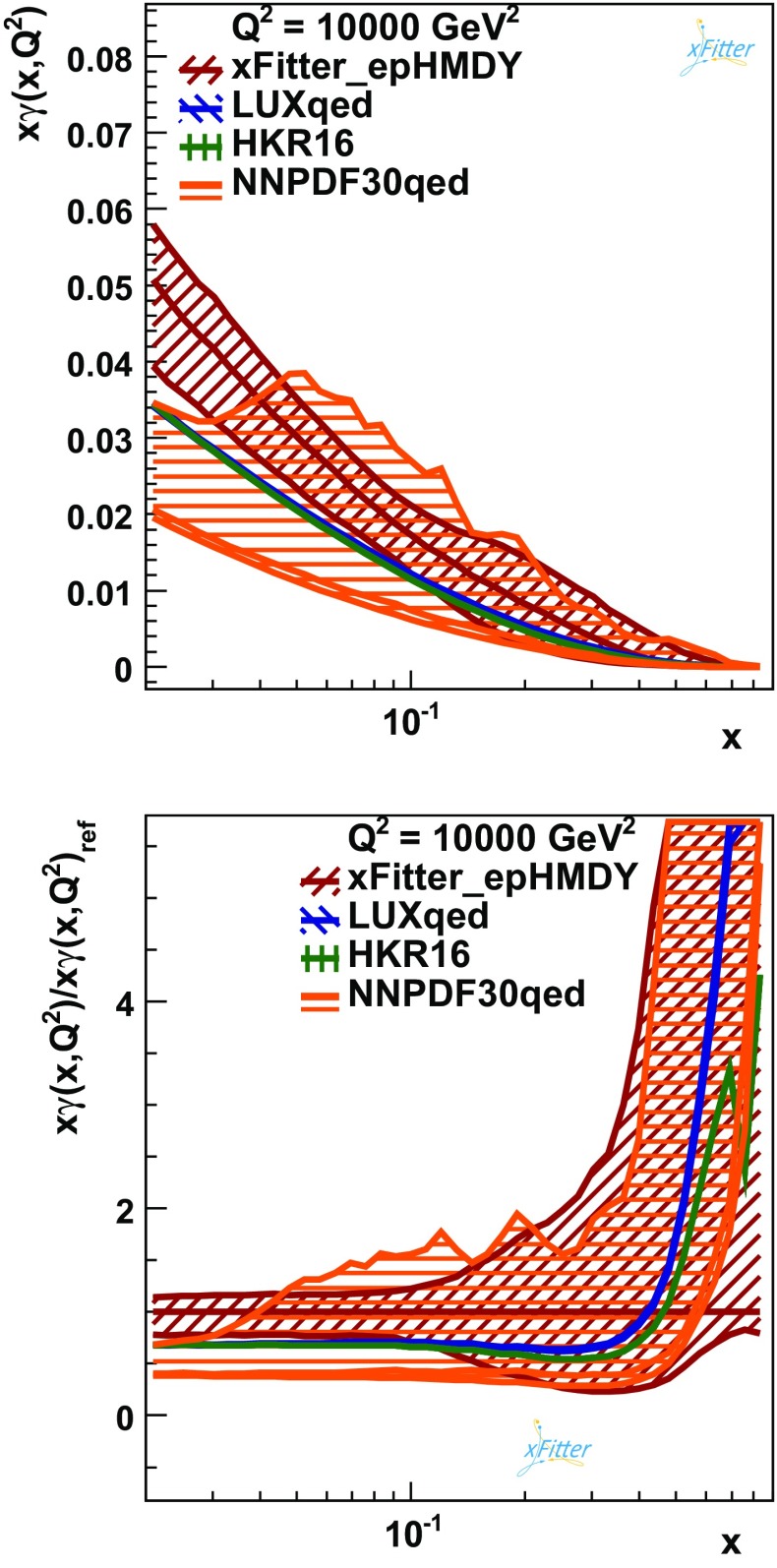
*Upper plot* Comparison between the photon $x\gamma (x,Q^2)$ at $Q^2=10^4$ GeV$^2$ from the present NNLO analysis (xFitter_epHMDY) with the corresponding results from NNPDF3.0QED, LUXqed and HKR16. *Lower plot* The same comparison, now with the results normalised to the central value of xFitter_epHMDY. For the present fit, the PDF uncertainties are shown at the 68% CL obtained from the MC method, while model and parametrisation uncertainties are discussed below. For HKR16 only the central value is shown, while for LUXqed the associated PDF uncertainty band [7] is included

### The photon PDF from LHC high-mass DY data

In Fig. 6, the photon PDF, $x\gamma (x,Q^2)$, is shown at $Q^2=10^4$ GeV$^2$, and it is compared to the corresponding LUXqed, HKR16 and NNPDF3.0QED results. In the upper plot the comparison is presented in an absolute scale, while in the lower plot the ratio of different results normalised to the central value of the fit is shown. For the present fit, xFitter_epHMDY, the experimental PDF uncertainties at the 68% confidence level (CL) are obtained from the Monte Carlo method, while model and parametrisation uncertainties are discussed below.

Likewise, the NNPDF3.0QED PDF set is shown the 68% CL uncertainty band, while for LUXqed the associated PDF uncertainty band is computed according to the prescription of Ref. [7].

For HKR16, only the central value is available. The *x*-range in Fig. 6 has been restricted to the region $0.02 \le x \le 0.9$, since beyond that region there is only limited sensitivity to $x\gamma (x,Q^2)$.

Figure 6 shows that for $x\ge 0.1$ the four determinations of the photon PDF are consistent within PDF uncertainties. For smaller values of *x*, the photon PDF from LUXqed and HKR16 is somewhat smaller than xFitter_epHMDY, but still in agreement at the 2$\sigma$ level. This agreement is further improved if the PDF uncertainties in xFitter_epHMDY arising from variations of the input parametrisation are added to experimental uncertainties, as discussed in Sect. 4.3. Moreover, the results of this work and NNPDF3.0QED agree at the 68% CL for $x\ge 0.03$, and the agreement extends to smaller values of *x* once the parametrisation uncertainties in xFitter_epHMDY are accounted for. The LUXqed and the HKR16 calculations of $x\gamma (x,Q^2)$ are very close to each other across the entire range of *x*.

Figure 6 shows that for $0.04 \le x \le 0.2$ the present analysis exhibits smaller PDF uncertainties as compared to those from NNPDF3.0QED. Indeed, the experimental uncertainty on the xFitter_epHMDY turns out to be at the $\sim$30% level for $x\le 0.1$. At larger *x* it increases rapidly specially in the positive direction. The reason for this behaviour at large *x* can be understood by recalling that variations of $x\gamma (x,Q^2)$ in the negative direction are constrained by positiveness. The limited sensitivity of the ATLAS data does not allow a determination of $x\gamma (x,Q^2)$ with uncertainties competitive with those of LUXqed, which are at the few percent level.

It is also interesting to assess the impact of the high-mass Drell–Yan 8 TeV measurements on the light quark and gluon PDFs. For this purpose, the fits have been repeated freezing the photon PDF to the xFitter_epHMDY shape. This is necessary because HERA inclusive data alone, which are the benchmark for this comparison, have no sensitivity to the photon PDF. This way, a meaningful comparison between the quark and gluon PDFs from a HERA-only baseline and the HERA + HMDY fit can be performed.

**Fig. 7:**
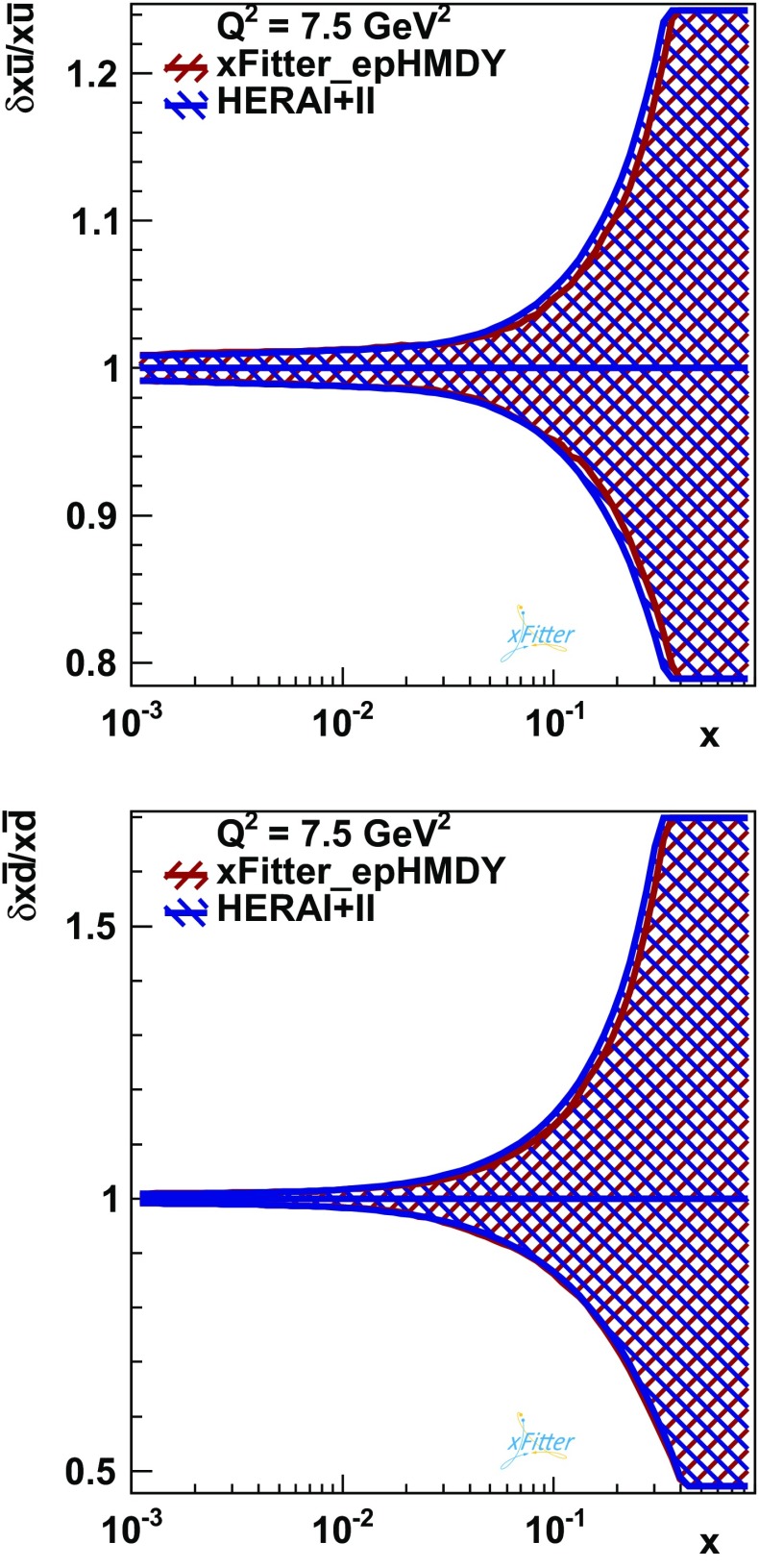
The impact of the ATLAS high-mass 8 TeV Drell–Yan measurements on the $x\bar{u}$ and $x\bar{d}$ sea-quark PDFs at the input parametrisation scale $Q^2=7.5$ GeV$^2$. The results are shown normalised to the central value of xFitter_epHMDY.

This comparison is shown in Fig. 7 for the up and down antiquarks $x\bar{u}(x,Q^2)$ and $x\bar{d}(x,Q^2)$, for which the effect of the high-mass Drell–Yan data is expected to be most pronounced, since HERA inclusive cross sections provide little information on quark flavour separation. In Fig. 7, the $x\bar{u}(x,Q^2)$ and $x\bar{d}(x,Q^2)$ together with the associated MC uncertainties have been computed at the initial parametrisation scale of $Q^2=7.5$ GeV$^2$ and are shown as ratios to the central value of the xFitter_epHMDY fit. The modifications in the medium and large-*x* antiquark distributions from the high-mass DY data are rather moderate. It has been verified that the same conclusions can be derived from fits obtained by switching off the QED effects for both the HERA-only fits and the HERA+HMDY fits. Therefore, while the ATLAS high-mass Drell–Yan measurements have a significant constraint on the photon PDF, their impact on the quark and gluon PDFs is moderate.

### Robustness and perturbative stability checks

Following the presentation of the main result of this work, the xFitter_epHMDY determination of the photon PDF $x\gamma (x,Q^2)$, the robustness of this determination with respect to a number of variations is assessed. Firstly, variations in the values of the input physical parameters, such as $\alpha _s$ or the charm mass are explored. Secondly, variations of the choices made for the PDF input parametrisation are considered. Finally, variations associated to different methodological choices in the fitting procedure are quantified. In each case, one variation at a time is performed and compared with the central value of $x\gamma (x,Q^2)$ and its experimental PDF uncertainties computed using the Monte Carlo method.

**Fig. 8:**
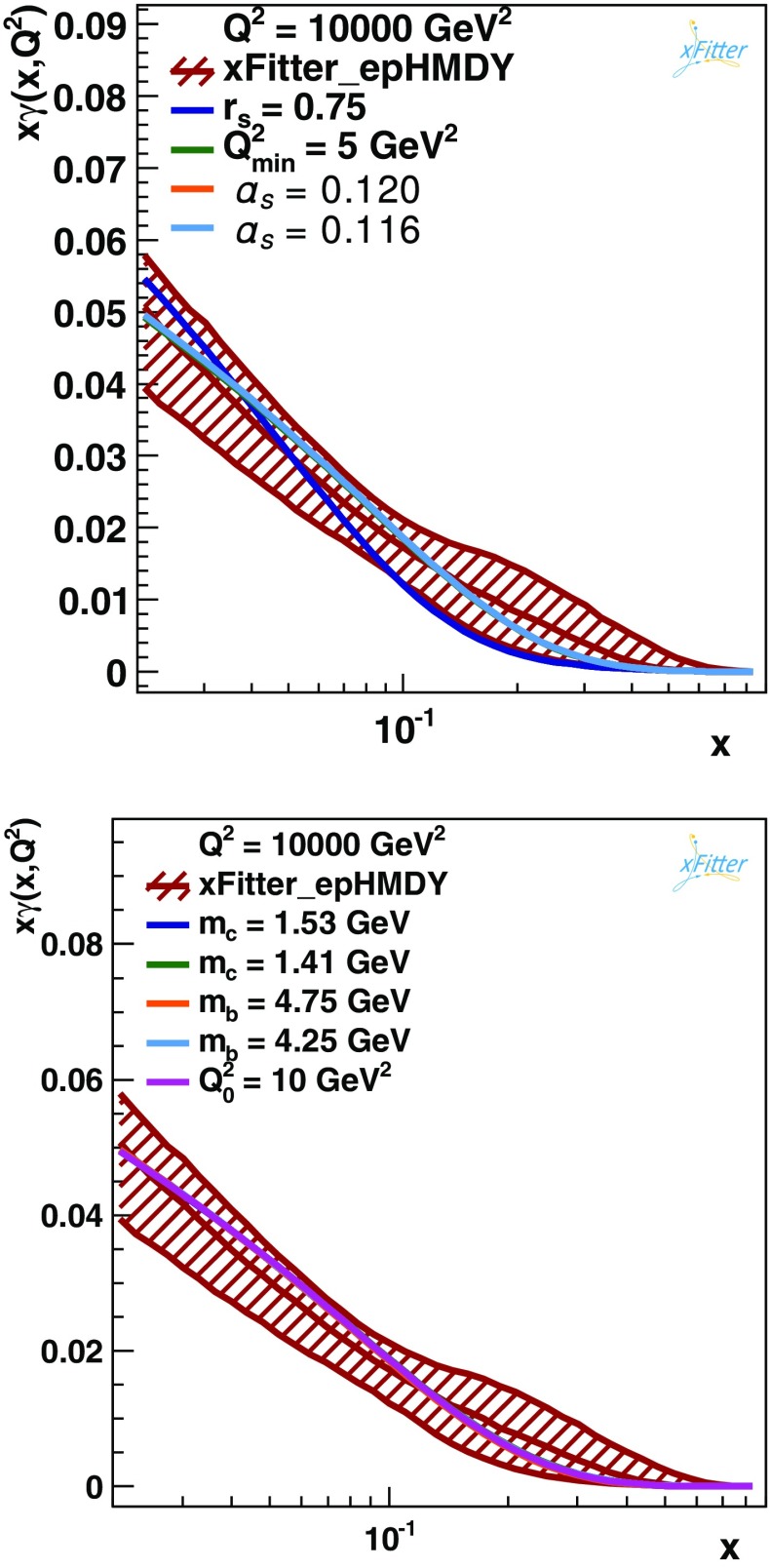
Comparison between the baseline determination of $x\gamma (x,Q^2)$ at $Q^2=10^4$ GeV$^2$ in the present analysis, xFitter_epHMDY, with the central value of a number of fits for which one input parameter has been varied. The following variations have been considered: $r_s=0.75$, $Q^2_\mathrm{min}=5$ GeV$^2$, $\alpha _s=0.116$ and 0.118 (*upper plot*); and $m_c=1.41$ and 1.53 GeV, $m_b=4.25$ and 4.75 GeV, and $Q_0^2=10$ GeV$^2$ (*lower plot*). The *curves* are indistinguishable because they overlap due to their negligible impact on photon PDF fit. Only the impact of the variation of the strange fraction assumption is visible by eye. See text for more details of these variations

First the impact of uncertainties associated to either the choice of input physical parameters or of specific settings adopted in the fit is considered. Figure 8 shows the comparison between the xFitter_epHMDY determination of $x\gamma (x,Q^2)$ at $Q^2=10^4$ GeV$^2$, including the experimental MC uncertainties, with the central value of those fits for which a number of variations have been performed. Specifically:

- The strong coupling constant is varied by $\delta \alpha _s=\pm 0.002$ around the central value.
- The ratio of strange to non-strange light quark PDFs is decreased to $r_s=0.75$ instead of $r_s=1$.
- The value of the charm mass is varied between $m_c=1.41$ GeV and $m_c=1.53$ GeV, and that of the bottom mass between $m_b=4.25$ GeV and $m_b=4.75$ GeV.
- The minimum value $Q_\mathrm{min}^2$ of the fitted data is decreased down to 5 GeV$^2$.
- The input parametrisation scale $Q_0^2$ is raised to 10 GeV$^2$ as compared to the baseline value of $Q_0^2=7.5~$GeV$^2$.

The results of Fig. 8 highlight that in all cases effect of the variations considered here is contained within (and typically much smaller than) the experimental PDF uncertainty bands of the reference fit. The largest variation comes from the strangeness ratio $r_s$, where the resulting central value turns out to be at the bottom end of the PDF uncertainty band for $x\ge 0.1$.

**Fig. 9:**
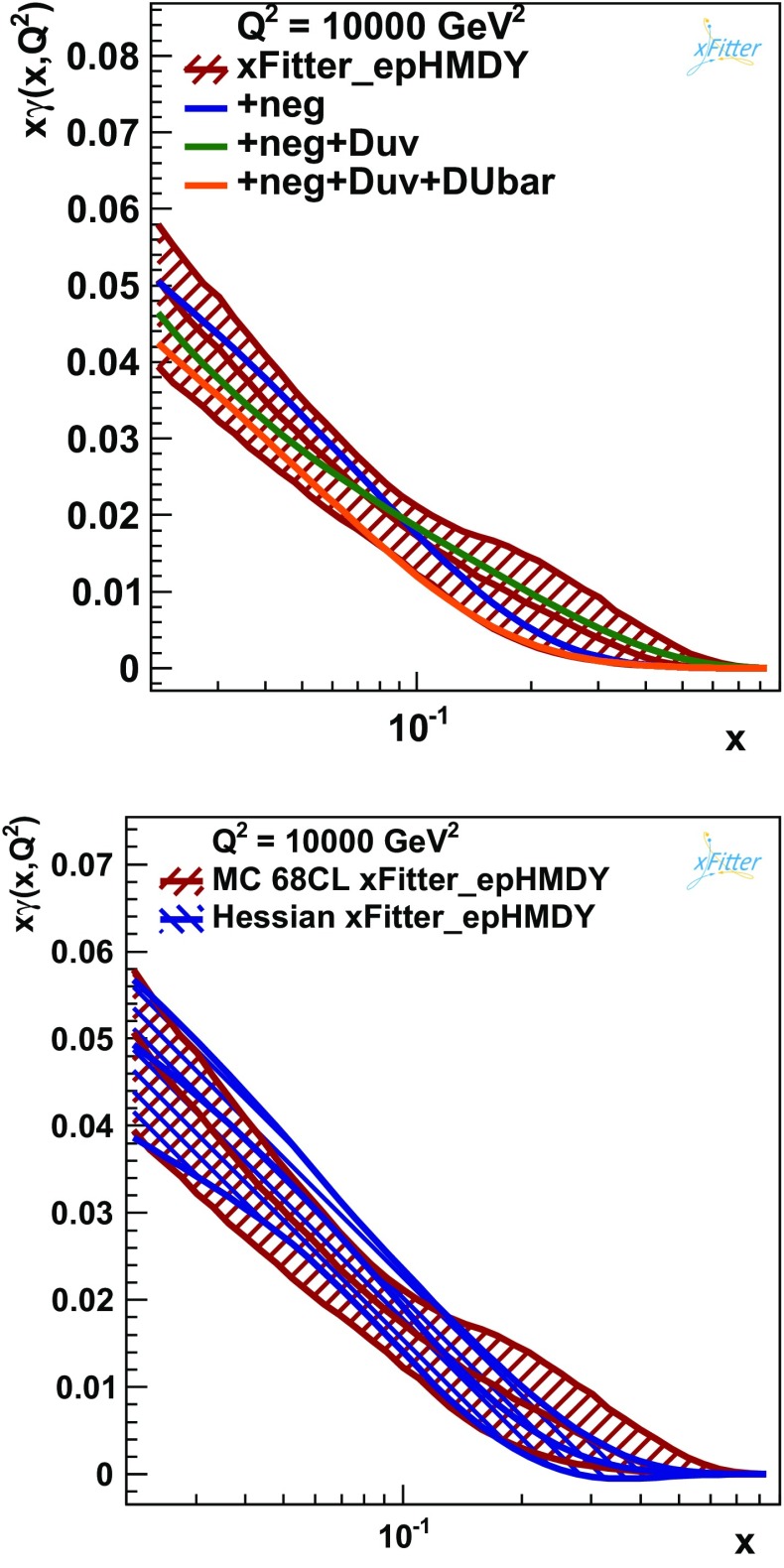
*Upper plot* The impact on the photon PDF $x\gamma (x,Q^2)$ from xFitter_epHMDY in fits where a number of additional free parameters are allowed in the PDF parametrisation Eq. (3). The parametrisation variations that have been explored are: more flexibility to the gluon distribution, allowing it to become negative (labeled by “$\mathrm{neg}$”), adding on top $D_{u_v}$, and then adding $D_{\bar{u}}+D_{\bar{d}}$. *Lower plot* Comparison between the xFitter_epHMDY determinations obtained with the Monte Carlo (baseline) and with the Hessian methods, where in both cases the PDF error band shown corresponds to the 68% CL uncertainties

Another important check of the robustness of the present determination of $x\gamma (x, Q^2)$ can be obtained by comparing the baseline fit with further fits where a number of new free parameters are allowed in the PDF parametrisation, in addition to those listed in Eq. (3). Figure 9 shows the impact of three representative variations (others have been explored, leading to smaller differences): more flexibility to the gluon distribution, allowing it to become negative at the initial scale (labeled by “$\mathrm{neg}$”), in addition to $D_{u_v}$, and then $D_{\bar{u}}+D_{\bar{d}}$. As before, all variations are contained within the experimental PDF uncertainty bands, though the impact of the parametrisation variations is typically larger than that of the model variations: in the case of the $\mathrm{neg}+D_{\bar{u}}+D_{\bar{d}}$ variations, the central value is at the lower edge of the PDF uncertainty band in the entire range of *x* shown.

A cross-check of the robustness of the estimated experimental uncertainty of the photon PDF in this analysis is provided by the comparison of the Monte Carlo and Hessian methods. Figure 9 shows this comparison indicating a reasonable agreement between the two methods. In particular, the central values of the photon obtained with the two fitting techniques are quite similar to each other. As expected, the MC uncertainties tend to be larger than the Hessian ones, specially in the region $x\gtrsim 0.2$, indicating deviations with respect to the Gaussian behaviour of the photon PDF.

**Fig. 10:**
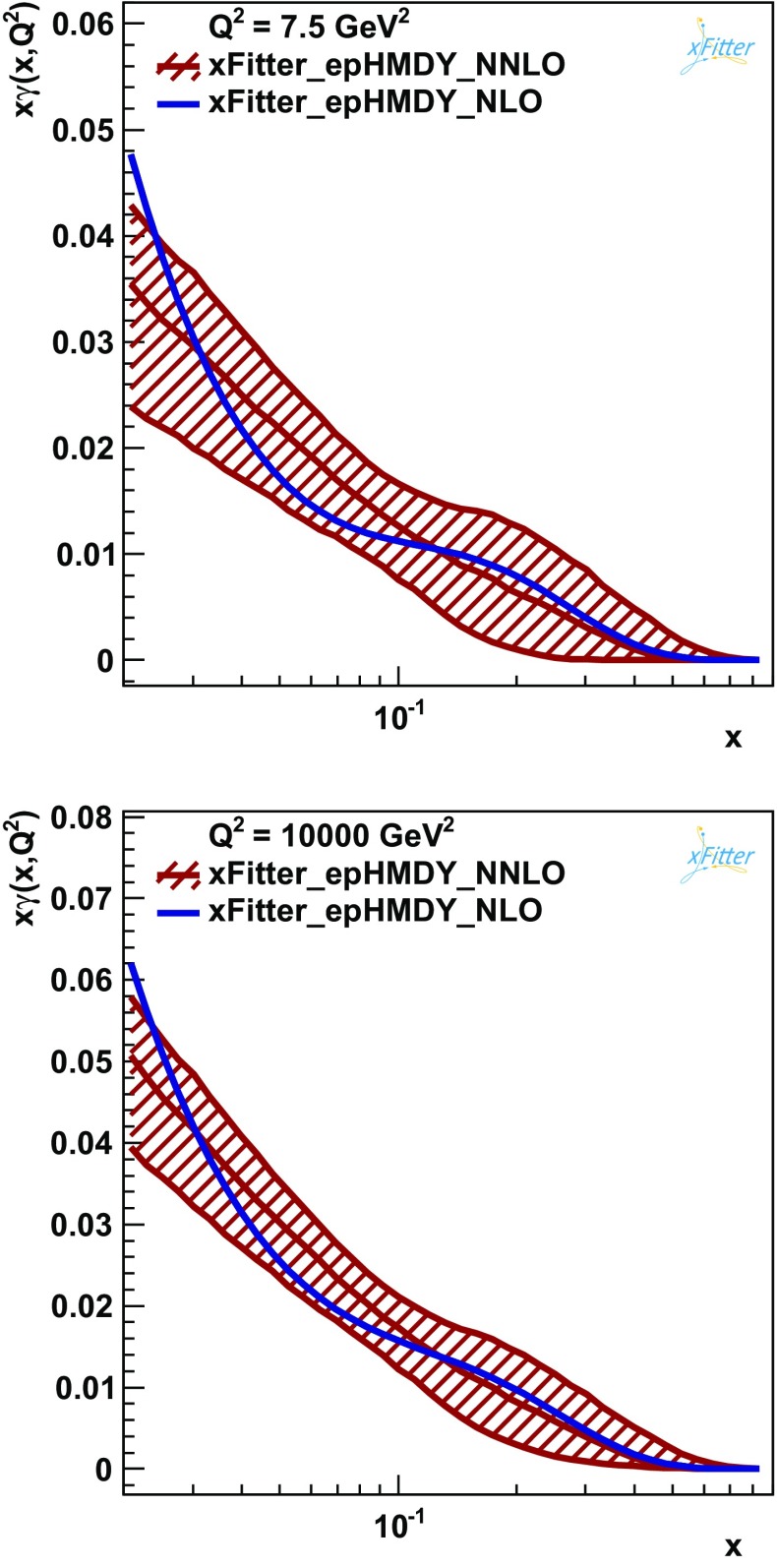
*Upper plot* comparison between the reference xFitter_epHMDY fit of $x\gamma (x,Q^2)$, based on NNLO QCD and NLO QED theoretical calculations, with the central value of the corresponding fit based on NLO QCD and QED theory, at $Q^2=7.5$ GeV$^2$. In the former case, only the experimental Monte Carlo PDF uncertainties are shown. *Lower plot* Same comparison, now presented at the higher scale of $Q^2=10^4$ GeV$^2$

To complete these studies, an interesting exercise is to quantify the perturbative stability of the xFitter_epHMDY determination of the photon PDF $x\gamma (x,Q^2)$ with respect to the inclusion of NNLO QCD corrections in the analysis. To study this, Fig. 10 shows a comparison between the baseline fit of $x\gamma (x,Q^2)$, based on NNLO QCD and NLO QED theoretical calculations, with the central value resulting from a corresponding fit based instead on NLO QCD and QED theory. In other words, the QED part of the calculations is identical in both cases. For the NNLO fit, only the experimental PDF uncertainties, estimated using the Monte Carlo method, are shown. From the comparison of Fig. 10, it is clear that the fit of $x\gamma (x,Q^2)$ exhibits a reasonable perturbative stability, since the central value of the NLO fit is always contained in the one-sigma PDF uncertainty band of the baseline xFitter_epHMDY fit. The agreement between the two fits is particularly good for $x\gtrsim 0.1$, where the two central values are very close to each other. This comparison is shown at low scale, $Q^2=7.5$ GeV$^2$ and high scales $Q^2=10^4$ GeV$^2$, indicating that perturbative stability is not scale dependent.

## Summary

In this work, a new determination of the photon PDF from a fit of HERA inclusive DIS structure functions supplemented by ATLAS data on high-mass Drell–Yan cross sections has been presented, based on the xFitter framework. As suggested by a previous reweighting analysis [9], this high-mass DY data provides significant constraints on the photon PDF, allowing a determination of $x\gamma (x, Q^2)$ with uncertainties at the 30% level for $0.02 \le x \le 0.1$. The results of the present study, dubbed xFitter_epHMDY, are in agreement and exhibit smaller PDF uncertainties that the only other existing photon PDF fit from LHC data, the NNPDF3.0QED analysis, based on previous LHC Drell–Yan measurements.

The results are in agreement within uncertainties with two recent calculations of the photon PDF, LUXqed and HKR16. For $x\ge 0.1$, the agreement is at the 1$\sigma$ level already including only the experimental MC uncertainties, while for $0.02 \ge x \ge 0.1$ it is important to account for parametrisation uncertainties. The findings indicate that a direct determination of the photon PDF from hadron collider data is still far from being competitive with the LUXqed and HKR calculations, which are based instead on precise measurements of the inclusive DIS structure functions of the proton.

The results of this study, which are available upon request in the LHAPDF6 format [43], have been made possible by a number of technical developments that should be of direct application for future PDF fits accounting for QED corrections. First of all, the full NLO QED corrections to the DGLAP evolution equations and the DIS structure functions have been implemented in the APFEL program. Moreover, our results illustrate the flexibility of the xFitter framework to extend its capabilities beyond the traditional quark and gluon PDF fits. Finally, the extension of aMCfast and APPLgrid to allow for the presence of photon-initiated channels in the calculations provided by MadGraph5_aMC@NLO, significantly streamlines the inclusion of future LHC measurements in PDF fits with QED corrections by consistently including diagrams with initial-state photons. All these technical improvements will certainly be helpful for future studies of the photon content of the proton.

## Figures and Tables

**Fig. 11:**
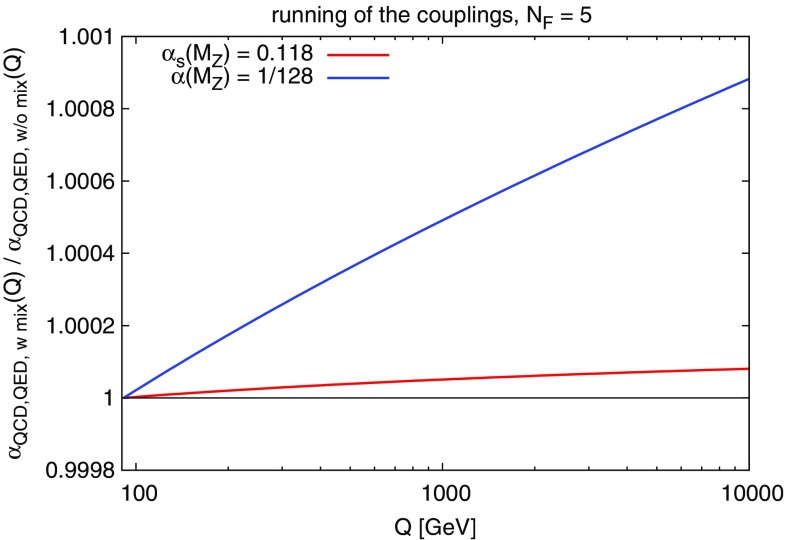
Comparison between the running with the scale *Q* of the QCD and QED couplings, $\alpha _s$ and $\alpha$, including or not the mixed terms in the corresponding $\beta$-functions. The *curves* are normalised to the result of the respective coupling running without the mixed terms included in the $\beta$ functions

**Fig. 12:**
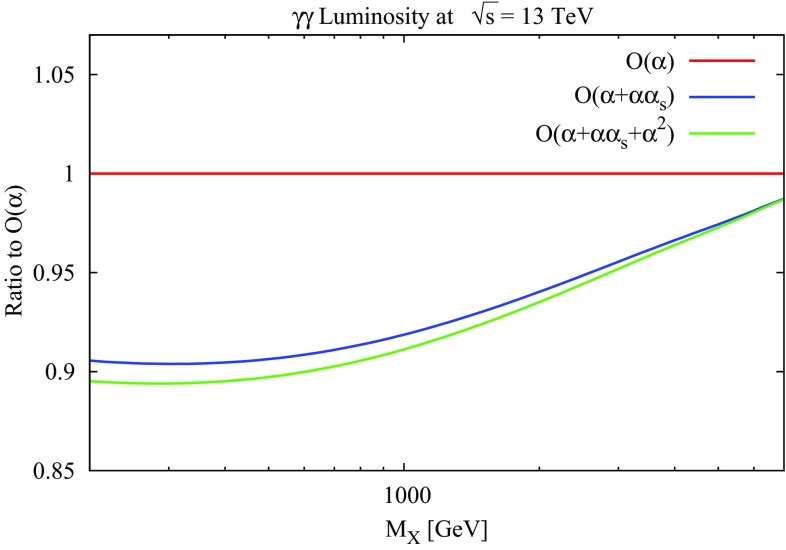
The photon–photon PDF luminosity $\mathscr {L}_{\gamma \gamma }$ at $\sqrt{s} = 13$ TeV as a function of the final-state invariant mass $M_X$. The results with the photon evolved with only the $\mathscr {O}(\alpha )$ corrections are compared with the corresponding results taking into account the $\mathscr {O}(\alpha +\alpha _s\alpha )$ corrections and the complete $\mathscr {O}(\alpha +\alpha _s\alpha +\alpha ^2)$ effects, normalised in all three cases to the $\mathscr {O}(\alpha )$ result. The calculation has been performed using the central value of the NNPDF3.0QED NLO fit

**Fig. 13:**
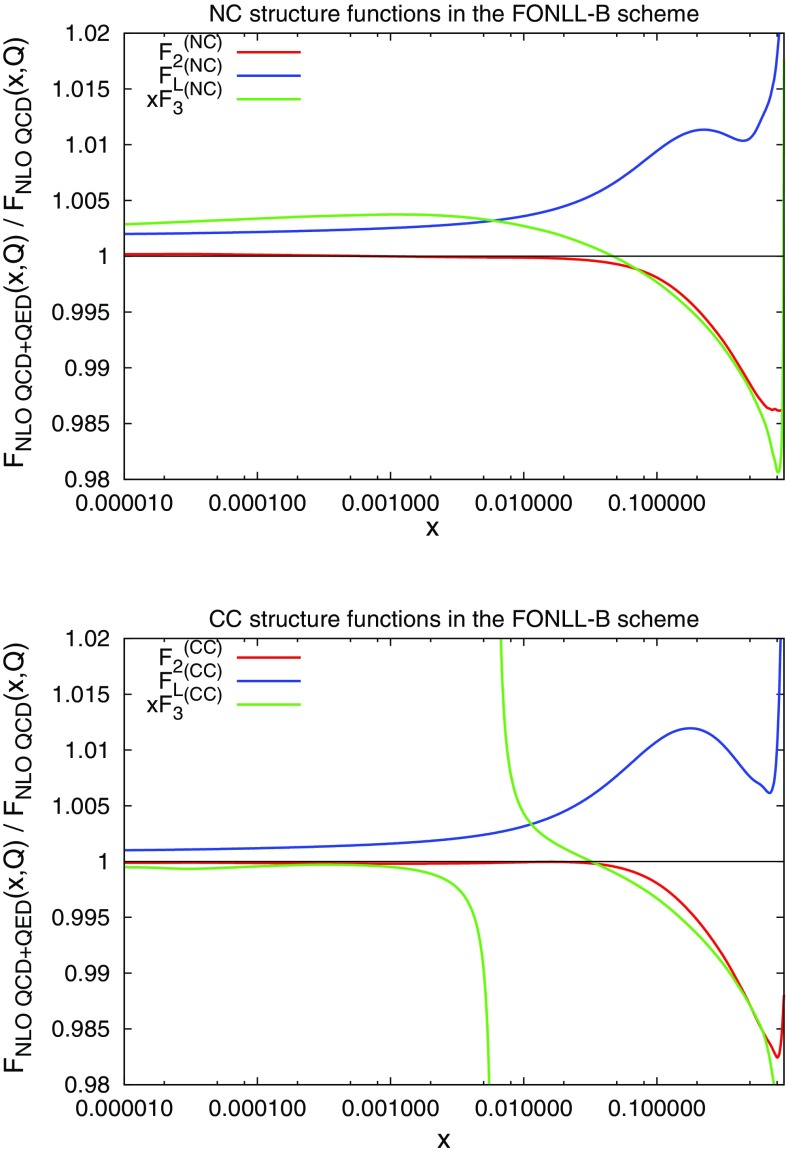
The effects of the NLO QED corrections on the neutral-current (*upper*) and charged-current (*lower plot*) DIS structure functions $F_2, F_L$ and $xF_3$, normalised to the pure QCD results. The calculation has been performed in the FONLL-B general-mass scheme using the central NNPDF3.0QED NLO set as input. Note that QED effects enter both via DGLAP evolution and the $\mathscr {O}(\alpha )$ DIS coefficient functions. The behaviour of $xF_3$ in the lower plot for $x\sim 0.007$ is explained by the fact that this structure function exhibits a node in that region

## Appendix A: Implementation of NLO QED corrections in APFEL

In this appendix, the details of the implementation of the combined NLO QCD + QED corrections in the APFEL program are presented. As discussed in Ref. [16], the implementation of the LO QED corrections to the DGLAP evolution equations presents many simplifications, in particular the fact that QED and QCD corrections do not mix and therefore the DGLAP equations, as well as the evolution equations for the running of $\alpha _s$ and $\alpha$, are decoupled. When considering NLO corrections, this property does not hold anymore, and QED and QCD contributions mix both in the DGLAP and in the coupling evolution equations. On top of this, NLO QED corrections induce the presence of diagrams with a real photon in the initial state, and these have to be consistently included in the computation of the DIS structure functions resulting in an explicit dependence on the photon PDF of these observables.

In the following, the discussion starts by discussing the generalisation of the equations for the running of the QCD and QED couplings, finding that the mixed QCD + QED terms have a negligible impact. Then the extension of the DGLAP evolution equations to account for the complete NLO QCD + QED effects is discussed. Finally, the modifications introduced by the NLO QED corrections in both the neutral-current and the charged-current DIS structure functions are discussed.

### Appendix A.1: Evolution of the couplings

As mentioned above, the NLO QCD+QED corrections induce the presence of mixed terms in the evolution equations of $\alpha _s$ and $\alpha$. In practice, the QCD $\beta$-function receives corrections proportional to $\alpha$ and, vice versa, the QED $\beta$-function receives corrections proportional to $\alpha _s$, in such a way that the coupling evolution equations read

A.1 $$\begin{aligned} \displaystyle \mu ^2\frac{\partial \alpha _s}{\partial \mu ^2}= & {} \displaystyle \beta ^\mathrm{QCD}(\alpha _s,\alpha ),\nonumber \\ \displaystyle \mu ^2\frac{\partial \alpha }{\partial \mu ^2}= & {} \displaystyle \beta ^\mathrm{QED}(\alpha _s,\alpha ). \end{aligned}$$

As a consequence, these evolution equations form a set of coupled differential equations. Up to three loops (i.e. NLO), the $\beta$-functions can be expanded as

A.2 $$\begin{aligned} \beta ^\mathrm{QCD}(\alpha _s,\alpha )= & {} -\alpha _s \bigg [\displaystyle \beta _0^{(\alpha _s)} \left( \frac{\alpha _s}{4\pi }\right) +\beta _1^{(\alpha _s\alpha )} \left( \frac{\alpha _s}{4\pi }\right) \left( \frac{\alpha }{4\pi }\right) \nonumber \\&+\displaystyle \beta _1^{(\alpha _s^2)}\left( \frac{\alpha _s}{4\pi }\right) ^2+\cdots \bigg ] \end{aligned}$$

and

A.3 $$\begin{aligned} \beta ^\mathrm{QED}(\alpha _s,\alpha )= & {} -\alpha \bigg [\displaystyle \beta _0^{(\alpha )}\left( \frac{\alpha }{4\pi }\right) +\beta _1^{(\alpha \alpha _s)}\left( \frac{\alpha }{4\pi }\right) \left( \frac{\alpha _s}{4\pi }\right) \nonumber \\&+\displaystyle \beta _1^{(\alpha ^2)}\left( \frac{\alpha }{4\pi }\right) ^2 +\cdots \bigg ], \end{aligned}$$

where the mixed terms, $\beta _1^{(\alpha _s\alpha )}$ and $\beta _1^{(\alpha \alpha _s)}$, and the pure NLO QED term, $\beta _1^{(\alpha ^2)}$, can be found in Ref. [44]. Taking into account a factor 4 due to the different definitions of the expansion parameters, one finds

A.4 $$\begin{aligned}&\displaystyle \beta _1^{(\alpha _s\alpha )} = -2\sum _{i=1}^{n_f}e_q^2,\nonumber \\&\displaystyle \beta _1^{(\alpha \alpha _s)} = -\frac{16}{3}N_c\sum _{i=1}^{n_f} e_q^2,\nonumber \\&\displaystyle \beta _1^{(\alpha ^2)} = -4\left( n_l+N_c\sum _{i=1}^{n_f} e_q^2\right) , \end{aligned}$$

where $N_c=3$ is the number of colours, $e_q$ is the electric charge of the quark flavour *q*, and $n_f$ and $n_l$ are the number of active quark and lepton flavours, respectively.

Equation (A.1) can be written in vectorial form:

A.5 $$\begin{aligned} \mu ^2\frac{\partial {\varvec{\alpha }}}{\partial \mu ^2} = {\varvec{\beta }}\left( {\varvec{\alpha }}(\mu )\right) , \end{aligned}$$

with

A.6 $$\begin{aligned} {\varvec{\alpha }} = {\alpha _s \atopwithdelims ()\alpha }\quad \text {and}\quad {\varvec{\beta }} = {\beta ^\mathrm{QCD} \atopwithdelims ()\beta ^\mathrm{QED}}. \end{aligned}$$

Equation (A.5) is an ordinary differential equation that can be numerically solved using, for example, Runge–Kutta methods.

The first two terms in Eq. (A.4) are responsible for the coupling of the evolution of $\alpha _s$ and $\alpha$, and thus they introduce a complication that affects both the implementation and the performance of the code. One can then ask what is the effect of their presence and whether their removal makes a substantial difference.

Figure 11 shows the comparison between the evolution at NLO of both couplings $\alpha _s$ and $\alpha$ including and excluding the mixed terms in the respective $\beta$-functions. The evolution is performed between the *Z* mass scale $M_Z$ and 10 TeV with 5 active quark flavours and 3 active lepton flavours and uses as boundary conditions $\alpha _s(M_Z) = 0.118$ and $\alpha (M_Z) = 1/128$. The two curves in Fig. 11 are normalised to the respective curves without mixed terms. It is clear that the mixed terms lead to tiny relative differences that are at most of $\mathscr {O}(10^{-4})$ at 10 TeV for $\alpha _s$ and $\mathscr {O}(10^{-3})$ at the same scale for $\alpha$. Thus it is safe to conclude that the mixed terms in the $\beta$-functions have a negligible effect on the evolution of the couplings and thus they are excluded to simplify the code and to improve the performance without introducing any significant inaccuracy.

### Appendix A.2: PDF evolution with NLO QED corrections

Next the implementation of the full NLO QCD+QED corrections to the DGLAP evolution equations is considered. The discussion is limited to consider of the photon PDF only, while the possible presence of the lepton PDFs is not addressed here. The first step towards an efficient implementation of the solution of the DGLAP equations in the presence of QED corrections is the adoption of a suitable PDF basis that diagonalises the splitting function matrix, decoupling as many equations as possible. Such a basis was already introduced in Appendix A of Ref. [45] and will be used also here. Excluding the lepton PDFs, this basis contains 14 independent PDF combinations and reads

A.7 $$\begin{aligned}&{\texttt { 1}:\, }g , \nonumber \\&{\texttt { 2} :\, }\gamma ,\nonumber \\&{\texttt { 3} :\, }\displaystyle \Sigma = \Sigma _u + \Sigma _d, \quad {\texttt {9} :\, }\displaystyle V =V_u + V_d,\nonumber \\&{\texttt { 4} : \,} \displaystyle \Delta _\Sigma = \Sigma _u - \Sigma _d, \quad \displaystyle {\texttt {10} : } \Delta _V = V_u - V_d ,\nonumber \\&{\texttt { 5} :\, }T_1^u = u^+ - c^+ ,\quad {\texttt {11} : }V_1^u = u^- - c^- ,\nonumber \\&{\texttt { 6} :\, }T_2^u = u^+ + c^+ - 2t^+ , \quad {\texttt {12} :\, }V_2^u = u^- + c^- - 2t^- , \nonumber \\&{\texttt { 7} :\, }T_1^d = d^+ - s^+ , \quad {\texttt {13} : }V_1^d = d^- - s^- ,\nonumber \\&{\texttt { 8} :\, }T_2^d = d^+ + s^+ - 2b^+, \quad {\texttt {14} :\, }V_2^d = d^- + s^- - 2b^-, \end{aligned}$$

where $q^\pm = q\pm \overline{q}$ with $q = u,d,s,c,b,t$. In addition

A.8 $$\begin{aligned} \Sigma _u = u^++c^++t^+,&\quad V_u = u^-+c^-+t^-,\nonumber \\ \Sigma _d = d^++s^++b^+,&\quad V_d = d^-+s^-+b^-. \end{aligned}$$

The second step is the construction of the splitting function matrix that determines the evolution of each of the combinations listed in Eq. (A.7). To this end, the splitting function matrix *P* is split into a pure QCD term $\widetilde{P}$, which only depends on $\alpha _s$, and a mixed QCD+QED correction term $\overline{P}$, which instead contains contributions proportional to at least one power of the QED coupling $\alpha$. In practice, this means that

A.9 $$\begin{aligned} P = \widetilde{P} + \overline{P}, \end{aligned}$$

where the pure QCD term reads

A.10 $$\begin{aligned} \widetilde{P} = \alpha _s \mathscr {P}^{(1,0)} + \alpha _s^2 \mathscr {P}^{(2,0)}+\cdots , \end{aligned}$$

while the term containing the QED coupling is given by

A.11 $$\begin{aligned} \overline{P} = \alpha \mathscr {P}^{(0,1)} + \alpha _s\alpha \mathscr {P}^{(1,1)}+\alpha ^2 \mathscr {P}^{(0,2)} + \cdots . \end{aligned}$$

Note that in the r.h.s. of Eqs. (A.10) and (A.11) the convention of Refs. [46, 47] is followed to indicate the power of $\alpha _s$ and $\alpha$ that each splitting function multiplies.

The structure of the pure QCD splitting function matrix $\widetilde{P}$ as well as the first term in $\overline{P}$, which represents the pure LO QED correction, were already discussed in Ref. [45]. It is now necessary to analyse the structure of the two additional terms, namely $\mathscr {P}^{(1,1)}$ and $\mathscr {P}^{(0,2)}$. Starting with the $\mathscr {O}(\alpha _s\alpha )$ term, the resulting evolution equations read

A.12 $$\begin{aligned}&\displaystyle \left. \mu ^2\frac{\partial }{\partial \mu ^2} \begin{pmatrix} g\\ \gamma \\ \Sigma \\ \Delta _\Sigma \end{pmatrix} \right| _{\mathscr {O}(\alpha _s \alpha )}\nonumber \\&\quad = \displaystyle \begin{pmatrix} e_\Sigma ^2 \mathscr {P}^{(1,1)}_{gg} &{} e_\Sigma ^2 \mathscr {P}^{(1,1)}_{g\gamma } &{} \eta ^+\mathscr {P}^{(1,1)}_{gq} &{} \eta ^-\mathscr {P}^{(1,1)}_{gq} \\ e_\Sigma ^2 \mathscr {P}^{(1,1)}_{\gamma g} &{} e_\Sigma ^2 \mathscr {P}^{(1,1)}_{\gamma \gamma } &{} \eta ^+\mathscr {P}^{(1,1)}_{\gamma q} &{}\eta ^-\mathscr {P}^{(1,1)}_{\gamma q} \\ 2 e_\Sigma ^2 \mathscr {P}^{(1,1)}_{qg} &{} 2 e_\Sigma ^2 \mathscr {P}^{(1,1)}_{q\gamma } &{} \eta ^+\mathscr {P}^{+(1,1)} &{} \eta ^-\mathscr {P}^{+(1,1)}\\ 2 \delta _e^2 \mathscr {P}^{(1,1)}_{qg} &{} 2 \delta _e^2 \mathscr {P}^{(1,1)}_{q\gamma } &{}\eta ^-\mathscr {P}^{+(1,1)} &{}\eta ^+\mathscr {P}^{+(1,1)} \end{pmatrix}\nonumber \\&\qquad \otimes \begin{pmatrix} g\\ \gamma \\ \Sigma \\ \Delta _\Sigma \end{pmatrix}, \end{aligned}$$

A.13 $$\begin{aligned} \displaystyle \left. \mu ^2\frac{\partial }{\partial \mu ^2} \begin{pmatrix} V\\ \Delta _V \end{pmatrix} \right| _{\mathscr {O}(\alpha _s \alpha )}= \begin{pmatrix} \eta ^+\mathscr {P}^{-(1,1)} &{} \eta ^-\mathscr {P}^{-(1,1)} \\ \eta ^-\mathscr {P}^{-(1,1)} &{} \eta ^+\mathscr {P}^{-(1,1)} \end{pmatrix} \otimes \begin{pmatrix} V\\ \Delta _V \end{pmatrix},\nonumber \\ \end{aligned}$$

A.14 $$\begin{aligned} \displaystyle \left. \mu ^2\frac{\partial T^u_{1,2}}{\partial \mu ^2}\right| _{\mathscr {O}(\alpha _s \alpha )}= & {} \displaystyle e_u^2\mathscr {P}^{+(1,1)}\otimes T^u_{1,2},\nonumber \\ \displaystyle \left. \mu ^2\frac{\partial T^d_{1,2}}{\partial \mu ^2}\right| _{\mathscr {O}(\alpha _s \alpha )}= & {} \displaystyle e_d^2\mathscr {P}^{+(1,1)} \otimes T^d_{1,2} ,\nonumber \\ \displaystyle \left. \mu ^2\frac{\partial V^u_{1,2}}{\partial \mu ^2}\right| _{\mathscr {O}(\alpha _s \alpha )}= & {} \displaystyle e_u^2\mathscr {P}^{-(1,1)} \otimes V^u_{1,2} ,\nonumber \\ \displaystyle \left. \mu ^2\frac{\partial V^d_{1,2}}{\partial \mu ^2}\right| _{\mathscr {O}(\alpha _s \alpha )}= & {} \displaystyle e_d^2\mathscr {P}^{-(1,1)}\otimes V^d_{1,2}, \end{aligned}$$

where $\otimes$ indicates the Mellin convolution and where

A.15 $$\begin{aligned} e_{\Sigma }^{2}\equiv & {} \displaystyle N_c(n_ue_{u}^{2}+n_de_{d}^{2}),\nonumber \\ \delta _e^2\equiv & {} \displaystyle N_c(n_u e_u^2 -n_d e_d^2),\nonumber \\ \eta ^{\pm }\equiv & {} \displaystyle \frac{1}{2}\left( e_{u}^{2}\pm e_{d}^{2}\right) , \end{aligned}$$

with $e_u$ and $e_d$ the electric charges of the up- and down-type quarks, and $n_u$ and $n_d$ the number of up- and down-type active quark flavours (such that $n_u+n_d=n_f$).

Next the $\mathscr {O}(\alpha ^2)$ corrections as considered. The expressions of the splitting functions at this order have been presented in Ref. [47]. There are two relevant new features that distinguish these corrections from the $\mathscr {O}(\alpha )$ and the $\mathscr {O}(\alpha _s\alpha )$ ones. The first one is that, contrary to the other cases in which the electric charges appears to the second power at most, here they appear up to the fourth power. As a consequence, new couplings must be introduced:

A.16 $$\begin{aligned} e_{\Sigma }^4= & {} N_c(n_{u} e_u^4 + n_{d} e_d^4),\nonumber \\ \delta _e^4= & {} N_c(n_{u} e_u^4 - n_{d} e_d^4). \end{aligned}$$

The second feature is that the dependence on the electric charges of some of the $\mathscr {O}(\alpha ^2)$ splitting functions is not factorisable as was the case for all the $\mathscr {O}(\alpha )$ and $\mathscr {O}(\alpha _s\alpha )$ ones and therefore a distinction must be made between up- and down-type splitting functions. Taking into account these features, it is possible to show that the $\mathscr {O}(\alpha ^2)$ contributions to the DGLAP equations take the following form:

A.17 $$\begin{aligned}&\displaystyle \left. \mu ^2\frac{\partial }{\partial \mu ^2} \begin{pmatrix} g\\ \gamma \\ \Sigma \\ \Delta _\Sigma \end{pmatrix} \right| _{\mathscr {O}(\alpha ^2)}\nonumber \\&\quad = \displaystyle \frac{1}{2} \begin{pmatrix} 0 &{} 0 &{} 0 &{} 0 \\ 0 &{} 2e_\Sigma ^4 \mathscr {P}_{\gamma \gamma }^{(0,2)} &{} e_u^4 \mathscr {P}_{\gamma u}^{(0,2)} + e_d^4 \mathscr {P}_{\gamma d} &{} e_u^4 \mathscr {P}_{\gamma u}^{(0,2)} - e_d^4 \mathscr {P}_{\gamma d}^{(0,2)}\\ 0 &{} 4 e_\Sigma ^4 \mathscr {P}^{(0,2)}_{q\gamma } &{} e_u^4\mathscr {P}_{uu}^{+(0,2)} +e_d^4\mathscr {P}_{dd}^{+(0,2)}+2\eta ^+e_\Sigma ^2\mathscr {P}^{S(0,2)}_{qq} &{} e_u^4\mathscr {P}_{uu}^{+(0,2)}-e_d^4\mathscr {P}_{dd}^{+(0,2)} + 2\eta ^-e_\Sigma ^2\mathscr {P}^{S(0,2)}_{qq}\\ 0 &{} 4 \delta _e^4 \mathscr {P}^{(0,2)}_{q\gamma } &{} e_u^4\mathscr {P}_{uu}^{+(0,2)} -e_d^4\mathscr {P}_{dd}^{+(0,2)}+2\eta ^-\delta _e^2 \mathscr {P}^{S(0,2)}_{qq} &{} e_u^4\mathscr {P}_{uu}^{+(0,2)}+e_d^4\mathscr {P}_{dd}^{+(0,2)} + 2\eta ^+\delta _e^2 \mathscr {P}^{S(0,2)}_{qq} \end{pmatrix}\otimes \begin{pmatrix} g\\ \gamma \\ \Sigma \\ \Delta _\Sigma \end{pmatrix},\nonumber \\ \end{aligned}$$

A.18 $$\begin{aligned}&\displaystyle \left. \mu ^2\frac{\partial }{\partial \mu ^2} \begin{pmatrix} V\\ \Delta _V \end{pmatrix} \right| _{\mathscr {O}(\alpha ^2)}\nonumber \\&\quad = \frac{1}{2} \begin{pmatrix} e_u^4\mathscr {P}_{uu}^{-(0,2)}+e_d^4\mathscr {P}_{dd}^{-(0,2)} &{} e_u^4\mathscr {P}_{uu}^{-(0,2)}-e_d^4\mathscr {P}_{dd}^{-(0,2)} \\ e_u^4\mathscr {P}_{uu}^{-(0,2)}-e_d^4\mathscr {P}_{dd}^{-(0,2)} &{} e_u^4\mathscr {P}_{uu}^{-(0,2)}+e_d^4\mathscr {P}_{dd}^{-(0,2)} \end{pmatrix}\nonumber \\&\quad \otimes \begin{pmatrix} V\\ \Delta _V \end{pmatrix}, \end{aligned}$$

A.19 $$\begin{aligned} \displaystyle \left. \mu ^2\frac{\partial T^u_{1,2}}{\partial \mu ^2}\right| _{\mathscr {O}(\alpha ^2)}= & {} \displaystyle e_u^4\mathscr {P}_{uu}^{+(0,2)}\otimes T^u_{1,2} ,\nonumber \\ \displaystyle \left. \mu ^2\frac{\partial T^d_{1,2}}{\partial \mu ^2}\right| _{\mathscr {O}(\alpha ^2)}= & {} \displaystyle e_d^4\mathscr {P}_{dd}^{+(0,2)} \otimes T^d_{1,2},\nonumber \\ \displaystyle \left. \mu ^2\frac{\partial V^u_{1,2}}{\partial \mu ^2}\right| _{\mathscr {O}(\alpha ^2)}= & {} \displaystyle e_u^4\mathscr {P}_{uu}^{-(0,2)} \otimes V^u_{1,2} ,\nonumber \\ \displaystyle \left. \mu ^2\frac{\partial V^d_{1,2}}{\partial \mu ^2}\right| _{\mathscr {O}(\alpha ^2)}= & {} \displaystyle e_d^4\mathscr {P}_{dd}^{-(0,2)}\otimes V^d_{1,2}. \end{aligned}$$

It should be noted that, as compared to the expressions for $\mathscr {P}^{(0,2)}$ presented in Ref. [47], the electric charges have been factored out in such a way that the expressions of the splitting functions are either independent from the electric charges themselves or depend on them only through the ratio $e_\Sigma ^2/e_q^2$.

As an illustration, the effects of the $\mathscr {O}(\alpha _s\alpha )$ and $\mathscr {O}(\alpha ^2)$ corrections to the DGLAP evolution equations on the $\gamma \gamma$ luminosity at $\sqrt{s} = 13$ TeV are quantified. This luminosity is defined by

A.20 $$\begin{aligned} \mathscr {L}_{\gamma \gamma }(M_X) = \frac{1}{s}\int _{M_X^2/s}^1 \frac{\mathrm{{d}}x}{x} \gamma (x,M_X^2) \gamma \left( \frac{M_X^2}{xs},M_X^2\right) , \end{aligned}$$

as a function of the final-state invariant mass $M_X$. Figure 12 illustrates the behaviour of $\mathscr {L}_{\gamma \gamma }$ computed using the photon PDF from the NNPDF30QED NLO set as an input at $Q_0 = 1$ GeV and evolved to $Q=M_X$ including, on top of the pure QCD NLO evolution, the following corrections:

- the $\mathscr {O}(\alpha )$ corrections only,
- same as above, adding also the mixed $\mathscr {O}(\alpha _s\alpha )$ corrections, and
- the complete NLO QCD+QED corrections accounting for the $\mathscr {O}(\alpha +\alpha _s\alpha +\alpha ^2)$ effects.

The results are shown normalised to the predictions obtained with LO QED corrections only. It is clear that the $\mathscr {O}(\alpha _s\alpha )$ and $\mathscr {O}(\alpha ^2)$ corrections have a small but non-negligible impact on the $\gamma \gamma$-luminosity. In particular, these corrections suppress $\mathscr {L}_{\gamma \gamma }$ by around 10% at relatively small values of $M_X$, while the suppression gradually shrinks to 1–2% as $M_X$ increases. As expected, most of this effect comes from the $\mathscr {O}(\alpha _s\alpha )$ corrections, while the impact of the $\mathscr {O}(\alpha ^2)$ ones is substantially smaller. These corrections to the DGLAP evolution have more recently been implemented also in the QEDEVOL package [17] based on the QCDNUM evolution code [18]. APFEL and QEDEVOL have been found to be in excellent agreement.

### Appendix A.3: DIS structure functions

When considering NLO QCD + QED corrections to the DIS structure functions, it becomes necessary to include into the hard cross sections all the $\mathscr {O}(\alpha )$ diagrams where one photon is either in the initial state or emitted from an incoming quark (or possibly an incoming lepton). Such diagrams, being of purely QED origin, have associated coefficient functions that can easily be derived from the QCD expressions by properly adjusting the colour factors. This correspondence holds irrespective of whether mass effects are included.

The main complication of the inclusion of these corrections arises from their flavour structure. In fact, in the case of quarks the isospin symmetry is broken due to the fact that the coupling of the photon is proportional to the squared charge of the parton to which it couples (a quark or a lepton). In the following, the neutral-current (NC) case, where lepton and proton exchange a neutral boson $\gamma ^*/Z$, and the charged-current (CC) case, where instead lepton and proton exchange a charged *W* boson, are addressed separately.

First the $\mathscr {O}(\alpha )$ contributions to a generic NC structure function *F* are considered. Due to the fact that to this order there is no mixing between QCD and QED, such corrections can easily be derived from the $\mathscr {O}(\alpha _s)$ coefficient functions just by adjusting the colour factors by setting $C_F=T_R=1$ and $C_A=0$. Referring, e.g., to the expressions reported in Ref. [48], the coefficient functions become

A.21 $$\begin{aligned} \begin{array}{rcl} \displaystyle C_{i;q}^{(\alpha )} &{}=&{} \displaystyle \frac{C_{i;q}^{(\alpha _s)}}{C_F}\\ \displaystyle C_{i;\gamma }^{(\alpha )} &{}=&{} \displaystyle \frac{C_{i;g}^{(\alpha _s)}}{T_R} \end{array}\qquad i = 2,L,3. \end{aligned}$$

In order to construct the corresponding structure functions, considering that the coupling between a photon and a quark of flavour *q* is proportional to $e_q^2$, the electroweak couplings should be adjusted as follows:

A.22 $$\begin{aligned} \widetilde{B}_q= & {} B_qe_q^2\quad \text{ for }\quad F_2,F_L,\nonumber \\ \widetilde{D}_q= & {} D_qe_q^2\quad \text{ for }\quad F_3, \end{aligned}$$

where $B_q$ and $D_q$ are defined, e.g., in Ref. [49]. Following this prescription, it is possible to write the $\mathscr {O}(\alpha )$ contributions to the NC structure functions:

A.23 $$\begin{aligned} F_{2,L}^{\mathrm{NC},(\alpha )}= & {} \displaystyle x \sum _{q} \widetilde{B}_q\left[ C_{2,L;q}^{(\alpha )}\otimes (q+\overline{q}) + C_{2,L;\gamma }^{(\alpha )} \otimes \gamma \right] ,\nonumber \\ xF_3^{\mathrm{NC},(\alpha )}= & {} \displaystyle x \sum _{q} \widetilde{D}_q\left[ C_{3;q}^{(\alpha )}\otimes (q-\overline{q}) + C_{3;\gamma }^{(\alpha )} \otimes \gamma \right] .\nonumber \\ \end{aligned}$$

This structure holds for both massless and massive structure functions. This aspect is relevant to the construction of the FONLL general-mass structure functions.

For the CC case the procedure to obtain the expressions of the $\mathscr {O}(\alpha )$ coefficient functions is exactly the same as in the NC case (see Eq. (A.21)). However, this case is more complicated because the flavour structure of CC structure functions is more complex. Taking into account the presence of a factor $e_q^2$ every time that a quark of flavour *q* couples to a photon, the $\mathscr {O}(\alpha )$ corrections to the CC structure functions $F_2$ and $F_L$ for the production of a neutrino or an anti-neutrino take the form

A.24 $$\begin{aligned} F_{2,L}^{\mathrm{CC},\nu ,(\alpha )}= & {} \displaystyle x\sum _{\begin{array}{c} {U=u,c,t}\\ {D=d,s,b} \end{array}}|V_{UD}|^2\left[ C_{2,L;q}^{(\alpha )}\otimes \left( e_D^2D +e_U^2\overline{U}\right) +2 C_{2,L;\gamma }^{(\alpha )}\otimes \gamma \right] ,\nonumber \\ F_{2,L}^{\mathrm{CC},\overline{\nu },(\alpha )}= & {} \displaystyle x\sum _{\begin{array}{c} {U=u,c,t}\\ {D=d,s,b} \end{array}}|V_{UD}|^2\left[ C_{2,L;q}^{(\alpha )}\otimes \left( e_D^2\overline{D} +e_U^2U\right) +2 C_{2,L;\gamma }^{(\alpha )}\otimes \gamma \right] ,\nonumber \\ \end{aligned}$$

where $V_{UD}$ are the elements of the CKM matrix. The flavour structure of $F_3$ is instead slightly different:

A.25 $$\begin{aligned} xF_3^{\mathrm{CC},\nu ,(\alpha )}= & {} \displaystyle x\sum _{\begin{array}{c} {U=u,c,t}\\ {D=d,s,b} \end{array}}|V_{UD}|^2\left[ C_{3;q}^{(\alpha )}\otimes \left( e_D^2D -e_U^2\overline{U}\right) +2 C_{3;\gamma }^{(\alpha )}\otimes \gamma \right] ,\nonumber \\ xF_3^{\mathrm{CC},\overline{\nu },(\alpha )}= & {} \displaystyle x\sum _{\begin{array}{c} {U=u,c,t} \\ {D=d,s,b} \end{array}}|V_{UD}|^2\left[ C_{3;q}^{(\alpha )}\otimes \left( -e_D^2\overline{D} +e_U^2U\right) +2 C_{3;\gamma }^{(\alpha )}\otimes \gamma \right] .\nonumber \\ \end{aligned}$$

In order to simplify the implementation, it is advantageous to assume that, in these particular corrections, the CKM matrix is a $3 \times 3$ unitary matrix. Note, however, that the exact CKM matrix is still used in the QCD part of the structure functions. This approximation introduces an inaccuracy of the order of the QED coupling $\alpha$ times the value of the off-diagonal elements of the CKM matrix and therefore it is numerically negligible.

As an illustration of the impact of the $\mathscr {O}(\alpha )$ correction on the DIS structure functions, Fig. 13 shows the effect of introducing these contributions on top of the pure QCD computation at NLO. The plots are produced by evolving the NNPDF3.0QED NLO set from $Q_0=1$ GeV to $Q=100$ GeV including the full NLO QCD + QED corrections discussed in the previous section and using the resulting evolved PDFs to compute the NC (upper panel) and the CC (lower panel) DIS structure functions in the FONLL-B scheme, including the $\mathscr {O}(\alpha )$ corrections to the coefficient functions discussed above. The predictions are shown normalised to the pure QCD computation where the QED corrections are absent both in the evolution and in the computation of the structure functions.

It is clear that the impact of the full NLO QCD + QED corrections is pretty small especially in the low-*x* region where it is well below 1%. In the large-*x* region, instead, the presence of a photon-initiated contribution has a more significant effect because of the suppression of the QCD distributions (quarks and gluon) relative to the photon PDF and the impact of the QED corrections reaches the 2% level. It should be noticed that the behaviour around $x=10^{-2}$ of the CC $xF_3$ (green curve in the lower panel) is driven by a change of sign of the predictions so that the ratio diverges. Finally, we stress that the general features observed in Fig. 13 do not depend on the input PDF set. In particular, we have checked that the same picture holds using the xFitter_epHMDY PDF set presented in this work.

## Footnotes

1 Modified version of APPLgrid available at: https://github.com/scarrazza/applgridphoton.
